# The Human RNA Polymerase II-Associated Factor 1 (hPaf1): A New Regulator of Cell-Cycle Progression

**DOI:** 10.1371/journal.pone.0007077

**Published:** 2009-09-22

**Authors:** Nicolas Moniaux, Christophe Nemos, Shonali Deb, Bing Zhu, Irena Dornreiter, Michael A. Hollingsworth, Surinder K. Batra

**Affiliations:** 1 Department of Biochemistry and Molecular Biology, University of Nebraska Medical Center, Omaha, Nebraska, United States of America; 2 Howard Hughes Medical Institute, Department of Biochemistry, University of Medicine and Dentistry of New Jersey, Piscataway, New Jersey, United States of America; 3 Heinrich-Pette-Institute of Tumor Virology, Hamburg, Germany; 4 Eppley Institute, University of Nebraska Medical Center, Omaha, Nebraska, United States of America; Health Canada, Canada

## Abstract

**Background:**

The human PAF (hPAF) complex is part of the RNA polymerase II transcription apparatus and regulates multiple steps in gene expression. Further, the yeast homolog of hPaf1 has a role in regulating the expression of a subset of genes involved in the cell-cycle. We therefore investigated the role of hPaf1 during progression of the cell-cycle.

**Methodology/Findings:**

Herein, we report that the expression of hPaf1, a subunit of the hPAF complex, increases with cell-cycle progression and is regulated in a cell-cycle dependant manner. hPaf1 specifically regulates a subclass of genes directly implicated in cell-cycle progression during G1/S, S/G2, and G2/M. In prophase, hPaf1 aligns in filament-like structures, whereas in metaphase it is present within the pole forming a crown-like structure, surrounding the centrosomes. Moreover, hPaf1 is degraded during the metaphase to anaphase transition. In the nucleus, hPaf1 regulates the expression of cyclins A1, A2, D1, E1, B1, and Cdk1. In addition, expression of hPaf1 delays DNA replication but favors the G2/M transition, in part through microtubule assembly and mitotic spindle formation.

**Conclusion/Significance:**

Our results identify hPaf1 and the hPAF complex as key regulators of cell-cycle progression. Mutation or loss of stoichiometry of at least one of the members may potentially lead to cancer development.

## Introduction

The RNA polymerase II-associated factor (PAF) complex is a mediator of histone ubiquitinylation and methylation during the transcription process in yeast [1], [2], plants [3], and mammals [4]. The yeast Paf1 (yPaf1) complex binds to RNA polymerase II (RNAPII), coordinating co-transcriptional histone modifications such as histone H2B mono-ubiquitinylation and histone H3-Lys4, -Lys79 methylation and participates in transcription initiation and elongation [1], [5].

The transcriptional process is predominantly mediated by the activity of RNA polymerase II (RNAPII). As eukaryotic RNAPII cannot bind directly to DNA, the initiation of transcription depends on promoter recognition by general transcription factors (GTFs) [6], [7]. PAF complex is one of these factors [for review,[8], [9]]. The PAF complex directly interacts with RNA polymerase II and regulates multiple steps during gene expression [10], [11] including transcription [1], [2], [5], elongation [12], mRNA stability, RNA quality control [11], and RNA export to the cytoplasm [11], [12]. The first cDNA encoding a subunit of the hPAF complex, hPaf1 (AJ401156), was identified in our laboratory as a differentially expressed mRNA between the poorly differentiated human pancreatic tumor cell line, Panc1, and the well-differentiated cell line, CD11 [13]. Others identified the hPaf1 protein through co-purification with the parafibromin tumor suppressor protein, the human homologue of the yeast Cdc73 [4], [14]. The hCdc73 protein is the product of the *HRPT2* gene, a tumor suppressor involved in the hyperparathyroidism-jaw tumor syndrome (HPT-JT) [15], [16]. Several mutated forms of hCdc73 lack the ability to interact with other members of the hPAF complex. Interestingly, among the five members forming the yeast complex, only hCdc73, hPaf1, hCtr9, and hLeo1 were found as subunits of the human complex. Rtf1, present in the yeast complex, is not purified with the hPAF complex [4], [11]. Human PAF complex interacts with RNAPII and with a histone lysine methyl transferase (HKMTase) [4]. Woodard *et al.* showed that wild-type hCdc73 inhibited cyclin D1 expression, but not the mutated form of hCdc73 (parafibromin) [17]. In addition, Zhu et al [11] recently reported that the hPAF complex shares a novel higher eukaryotic subunit hSki8, common with the human SKI (hSKI) complex, whose down regulation results in a reduction of the cellular levels of other hPAF subunits.

In the present study, the role of hPaf1 was investigated during progression of the cell-cycle based on two sets of observations. First, three reports suggested a role for the yeast homolog of Paf1 in regulating the expression of a subset of genes involved in the cell-cycle [18], [19]. Second, we observed that asynchronously growing cells undergoing mitosis do not express hPaf1 along with other subunits. Here, we show that the expression of hPaf1 is temporally regulated during the cell-cycle, and it is a key regulator of cyclin expression.

## Results

### The expression of hPaf1 is regulated during cell cycle

**Figure 1:**
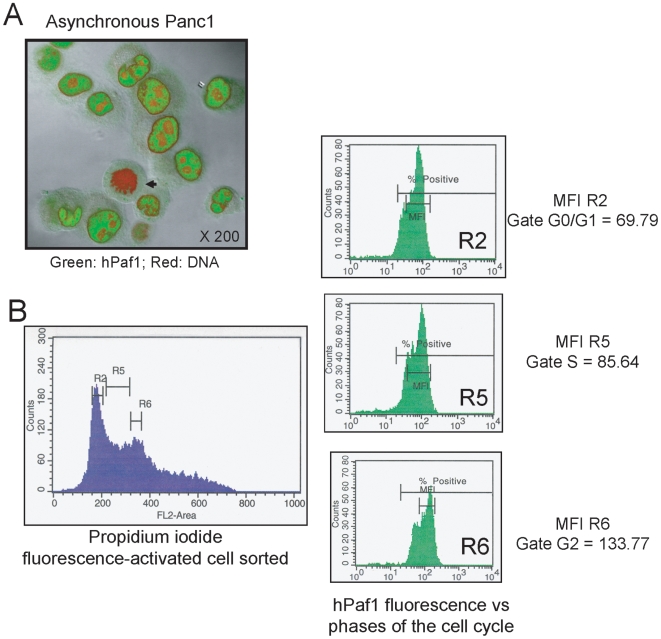
hPaf1 is differentially expressed during the cell cycle. A) The sub-cellular localization of hPaf1 was determined by confocal analysis of a population of asynchronously growing Panc1 cells that were labeled with FITC-conjugated anti-hPaf1 antibody (green) and counterstained with propidium iodide (red). hPaf1 was detected in the nucleus of the cells, but was absent from the heterochromatin and the nucleoli. hPaf1 was not detected in cells going through mitosis (black arrow). B). The FITC-labeled hPaf1 protein was quantified for each phase of the cell cycle. The mean of fluorescence detected was of 69.79, 85.64, and 133.77 for the cells in the G1-, S- and G2-phases, respectively.

When an asynchronously growing human pancreatic cancer cell, Panc1, population was observed for hPaf1 expression by confocal immunofluorescence microscopy, all cells undergoing mitosis showed a weak to no expression (Fig. 1A). Asynchronous cell cultures of Panc1 were further evaluated by staining with FITC conjugated anti-hPaf1 antibody, counterstaining with propidium iodide, and flow cytometric analysis of hPaf1 expression during different phases of the cell cycle (Fig. 1B). Twenty thousand cells per experiment were sorted in function of the cell cycle phase (G1, S, and G2) and each cell subpopulation further examined for hPaf1 expression. The relative fluorescence intensities were of 69.8, 85.6, and 133.8 for the cells in G1, S, and G2 phases, respectively. These data suggest that hPaf1 expression increases with the cell-cycle progression and that the expression of hPaf1 is regulated in a cell-cycle dependent manner.

**Figure 2:**
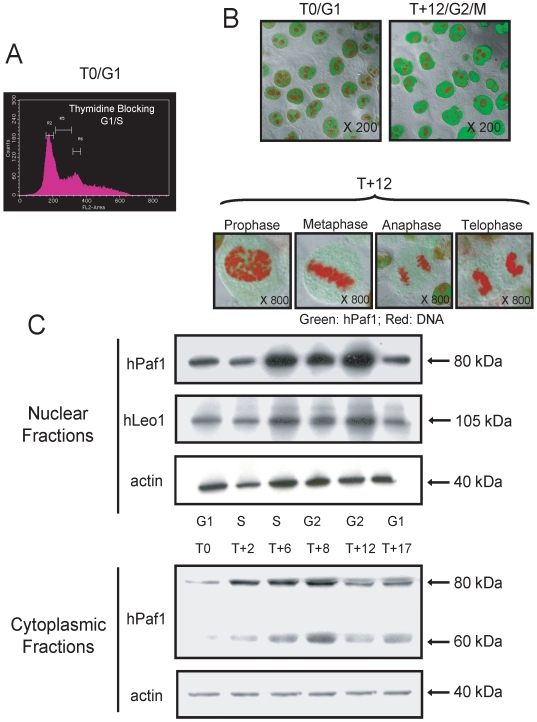
Localization of hPaf1 during the cell cycle in Panc1 cells. hPaf1 expression was monitored in a synchronously growing population of Panc1 cells. Panc1 cells were arrested at the G1/S boundary by double thymidine block, then released in normal medium containing serum (time 0 = T0) and analyzed every 2 h for 24 h by flow cytometry (after propidium iodide staining), confocal microscopy, and Western blotting of cytoplasmic and nuclear cell fractions. A) Eighty percent of the cells were blocked at the G1/S boundary. After release, the cells progressed into the cell cycle synchronously to reach a peak of mitosis after 10–12 h. Representative fields from the confocal analysis are presented in B). The cells were grown at low density on cover slips for 24 h, and arrested at the G1/S boundary by double thymidine incorporation. The acetone/methanol (1∶1)-fixed Panc1 cells were labeled using a FITC-conjugated anti-hPaf1 monoclonal antibody and counterstained by propidium iodide. The amount of fluorescence detected for the cells in T+12 (G2/M) was twice the level detected in T0 (G1/S). Up to 10 fields were monitored for each phase of the cell cycle. Cells in the prophase, metaphase, anaphase, and telophase showed very low to no expression of hPaf1. C) Nuclear and cytoplasmic protein extracts were prepared every 2 h and analyzed by immunoblotting using anti-hPaf1 and anti-hLeo1 antibodies. The JLA-20 antibody recognizing β actin was used as a loading control.

We further examined this hypothesis by monitoring hPaf1 expression in a synchronously growing population of Panc1 cells. Panc1 cells were arrested at the G1/S boundary by a double thymidine block. The cells were then released in normal medium containing serum (time 0 = T0) and analyzed every 2 hours for 24 hours by flow cytometry (after propidium iodide staining), confocal microscopy, and Western blotting of cytoplasmic and nuclear cell fractions. Results from flow cytometry showed that 80% of the cell population was in the G1/S phase just before release (Fig. 2A). The cells progressed into the cell cycle synchronously to reach a peak of mitosis after 10 to 12 hours(data not shown). The rates of cells with higher ploidies were noticeable by flow cytometry (reported to be of 8.5% for the Panc1 cell line [20]).

Immunofluorescence analysis showed the presence of hPaf1 in nuclei but not in the perinuclear heterochromatin or nucleoli (Fig. 2B). After 10 to 12 hours, the majority of cells entered mitosis. Interestingly, upon entry into mitosis, levels of hPaf1 dropped significantly to trace amounts during prophase. Western blot analysis confirmed these results. A single band of 80 kDa was detected in the nuclear fraction, while two bands of 60 and 80 kDa were observed in cytoplasmic extracts (Fig. 2C). The 60 kDa band was not detected in the cytoplasmic fraction of asynchronously growing cells. Both 60 and 80 kDa cytoplasmic bands increased from G1 to G2 with a detectable increase 2 hours after release, and a maximal increase after 8 h. Levels of hPaf1 dropped at 12 hours(mitosis), and re-appeared after 17 hours, when cells were back in the G1 phase. The nuclear band of 80 kDa presented a similar profile but with a detectable increase after 4 hours and a maximum at 12 h. The deduced molecular weight of hPaf1 is 59.9 kDa [13]. Therefore, our results suggest that the 60 kDa protein detected represents the hPaf1 precursor, which rapidly processes into an 80 kDa mature protein and is then transported to the nucleus and accumulates until the late G2 phase.

### hPaf1 is degraded during mitosis

**Figure 3:**
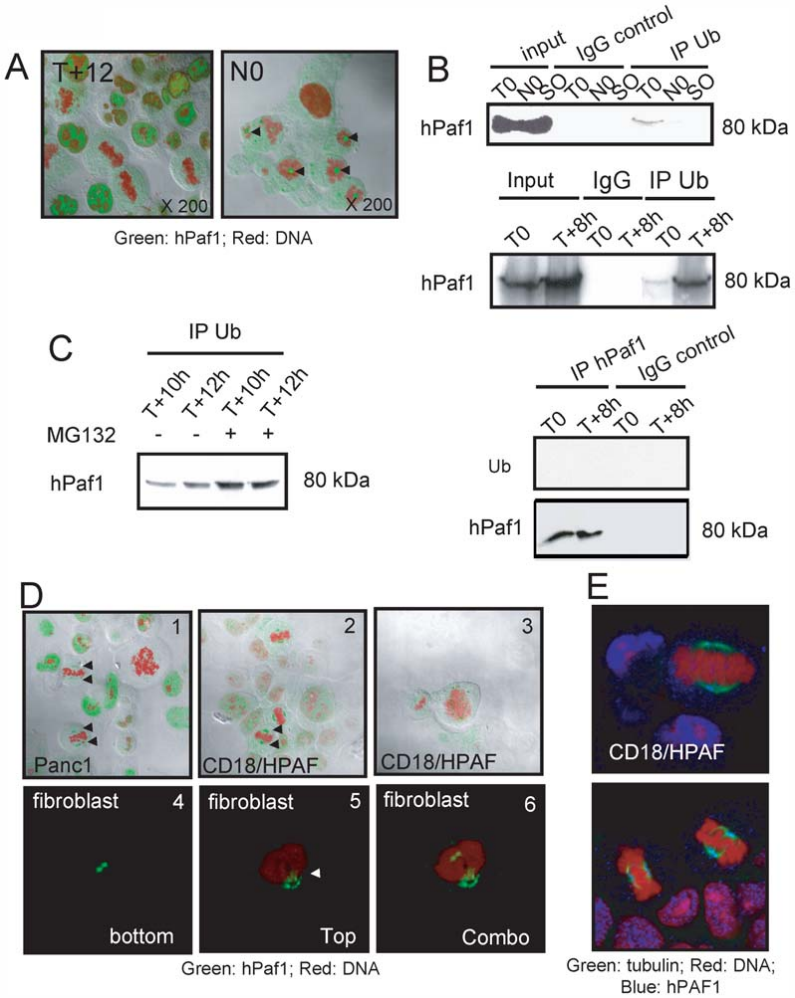
hPaf1 is ubiquitinylated and degraded at the metaphase/anaphase transition. Panc1 cells were grown at low density on cover slips for 24 h, and arrested at G1/S (T0) and G2/M (N0) boundaries by double thymidine and thymidine/nocodazole blocks, respectively. Acetone/methanol (1∶1)-fixed Panc1 cells were labeled using a FITC-conjugated anti-hPaf1 monoclonal antibody and counterstained by propidium iodide. Up to 10 fields were observed by confocal microscopy for cells after 12 h of double thymidine treatment release (T+12) and compared with 10 fields of thymidine/nocodazole treated cells just before release (N0). Representative pictures are presented in A). The black arrows indicate the inter-chromatine accumulation of hPaf1 in the prophase for the cells in N0; accumulation was not detected at T+12. B) Proteins from nuclear extracts performed at T0, T+8, N0, and S0 (shake off cells in N0) were immunoprecipitated using the rabbit polyclonal antibody anti-ubiquitin (Calbiochem), and immunoblotted with anti-hPaf1 antibody. An aliquot of the nuclear protein extracts was loaded as a control for hPaf1 expression (input), and immunoprecipitation was carried out using an isotype IgG control antibody as a negative control. C) hPaf1 degradation by the proteasome was confirmed using the proteasome inhibitor MG132. After double thymidine block, the cells were released either in ethanol control or MG132 (20 µM) for 12 h. Protein lysates were imunoprecipitated using the rabbit polyclonal antibody anti-ubiquitin (Calbiochem) and immunoblotted with anti-hPaf1 antibody. D) Panc1, CD18/HPAF, and human fibroblast cells were arrested at the G1/S boundary and released for 12 h to reach a maximum of mitosis. The cells were analyzed for hPaf1 expression and sub cellular localization by confocal. In the prophase, hPaf1 was found aligned on filament-like structures (D3) to be concentrated at the pole in the metaphase (D1, D2: black arrow). The Z-section (D4, D5, and D6) revealed that in the metaphase, hPaf1 formed a crown at both poles of the cells, potentially surrounding the centrosomes (white arrow). E) CD18/HPAF cells were arrested at the G1/S boundary and released for 12 h to reach a maximum of mitosis. The cells were analyzed for hPaf1 and tubulin expression and subcellular localization by confocal. In metaphase, hPaf1 co-localized with tubulin, confirming the centrosomal localization of hPaf1.

To precisely monitor the status of hPaf1 expression during mitosis, we compared ten different fields of confocal microscopy for Panc1 cells in T+12 (double thymidine block followed by 12 hours of release) and N0 (thymidine/nocodazole block) that synchronized the cells in late G2/early mitosis (checked by flow cytometry). Representative pictures of T+12 and N0 are depicted in Fig. 3A. Mitotic cells in N0 showed accumulation of hPaf1 in the cytoplasm and strong inter-chromatin labeling (black arrow). The hPaf1 protein appeared to concentrate between the condensed chromatin in metaphase. In comparison, cells in T+12 were slightly positive for cytoplasmic hPaf1, and no hPaf1 protein was observed within the condensed chromatin. As nocodazole is a microtubule-interfering agent that inhibits mitotic spindle formation, these results suggest that hPaf1 is shuttled or degraded at mitosis via tubulin filaments.

Since hPaf1 protein is overexpressed 30-fold in the Panc1 cells by amplification of its chromosomal locus [13], we evaluated its expression and localization in three other cell lines in which the gene is not amplified. CD18/HPAF (pancreatic cancer), MCF7 (breast cancer), and normal human fibroblast cell lines were arrested by double thymidine block at the G1/S boundary and released for 12 hours to reach a peak at mitosis. Analysis of hPaf1 expression during cell cycle progression was similar to that seen with Panc1, but at lower overall levels. Cells in mitosis were analyzed by confocal microscopy. The setting of the confocal was performed on the CD18/HPAF cells expressing the lowest level of hPaf1. Using these confocal parameters, the Panc1 cells were re-analyzed for hPaf1 expression/localization and compared with CD18/HPAF, MCF7, and human fibroblast cells. As depicted in Fig. 3D1, hPaf1 staining was detected for the cells in the metaphase. hPaf1 was concentrated at both cellular poles (Fig. 3D1, black arrows). Similar staining was observed for the CD18/HPAF (Fig. 3D2), MCF7, and human fibroblast (Fig. 3D6) cells. hPaf1 was detected in prophase showing a filament-like staining (Fig. 3D3), concentrated at the cellular poles in the metaphase (Fig. 3D2 and 3D6), for human fibroblast, MCF7, or CD18/HPAF cells. Z-sections of cells in the metaphase revealed that hPaf1 was forming a crown surrounding both centrosomes (Fig. 3D4, 3D5, and 3D6). The peri-centrosomal localization of hPaf1 was confirmed by tubulin/hPaf1 co-staining and confocal microscopy (Fig. 3DE). No staining was detected for any of the cells at anaphase or telophase. Although hPaf1 level of expression is 30-fold lower in the CD18/HPAF, and human fibroblast when compared with the Panc1 cells, hPaf1 concentration surrounding the centrosome was more intense in these two cell lines. This result raised the possibility that change in the stoichiometry of the hPAF complex following gene amplification or gene expression deregulation influenced the subcellular localization of hPAF complex, therefore modifying its function.

The aforementioned results suggested that hPaf1 is degraded at the transition from metaphase to telophase. To validate this hypothesis, protein lysates from cells at T0, T+8, N0, and shake-off (SO) conditions were incubated with rabbit polyclonal antibody against ubiquitin. Anti-ubiquitin immunoprecipitates were subjected to immunoblot analysis and associated hPaf1 was visualized using anti-hPaf1 antibody. An anti-hPaf1 reactive band with a molecular weight of 80 kDa was detected at very low levels at T0, but significant quantities were observed at T+8 (Fig. 3B). In contrast, hPaf1 was not detected at N0 or S0. Because hPaf1 pooled down using anti-ubiquitin antibody presented the same molecular weight as that in the input control, we concluded that hPaf1 is not directly ubiquitinylated, but associated with ubiquitinylated proteins. This hypothesis was validated by performing reverse immunoprecipitation. Anti-hPaf1 immunoprecipitates were subjected to immunoblot analysis and associated ubiquitinylated proteins were visualized using anti-ubiquitin antibody. No band was detected either at T0 or T+8 (Fig. 3B, lower panel). As control, the membrane was stripped and probed using the mouse monoclonal antibody, anti-hPaf1 (Fig. 3B, lower panel). To measure if hPaf1 is sensitive to proteasome activity, cytoplasmic protein lysates from synchronously growing Panc1 cells (at T+10 h and T+12 h to overlap the transition from G2 to M) were treated for 12 hours with the proteasome inhibitor MG132, then immunoprecipitated with the anti-ubiquitin antibody. Ethanol treated cells were used as negative control. There was a significant increase in the steady-state level of hPaf1 following MG132 treatment as compared to the ethanol control (Fig. 3C). Therefore, even if not directly ubiquitinylated, hPaf1 may be degraded by a mechanism dependent on the proteasome.

### hPaf1 modulates cell-cycle progression

**Figure 4:**
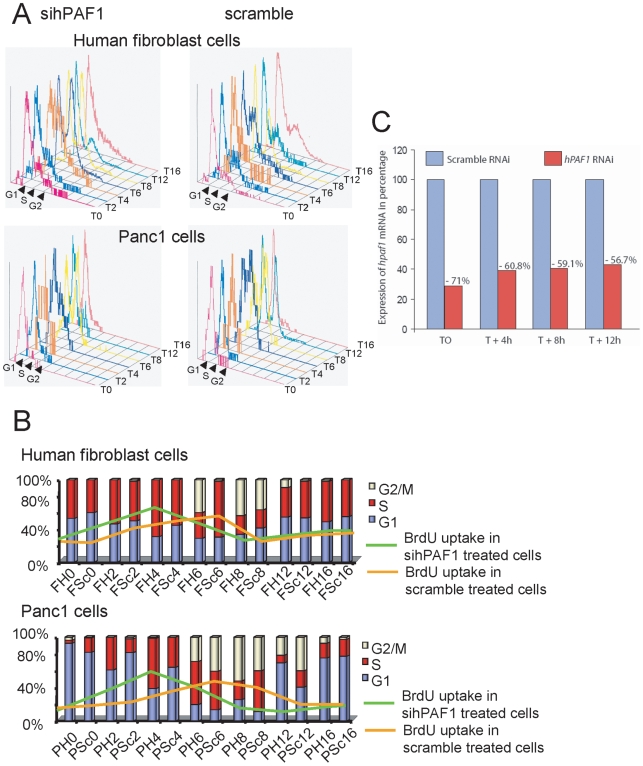
hPaf1 regulates the cell cycle. A) Panc1 and human normal fibroblast cells were transfected either using a hPaf1 or scramble siRNA, and synchronized at the G1/S boundary by double thymidine block. The cell cycle progression was measured by propidium iodide staining and BrdU uptake every 2 h for 24 h. The BrdU was added one hour before each time point. B) Bar diagram summarizing the results of the cell-cycle kinetic for both human fibroblast and Panc1 cells. FH: fibroblast cells knock down for hPaf1, FSc: fibroblast cells transfected with the scramble siRNA, PH: Panc1 cells knock down for hPaf1, PSc: Panc1 cells transfected with the scramble siRNA. The bars represent the percentage of cells in each phase of the cell-cycle measured by propidium iodide, and the lines represent the percentage of BrdU uptake for each time point. C) Suppression of hPaf1 expression by the fibroblast cells after transfection with siRNA oligonucleotide was measured by one-step real time RT-PCR using a TaqMan probe following the absolute standard curve method. Standard curves were established using hPaf1 and GAPDH mimics. Sequences and conditions used in this experiment are available upon request.

**Figure 5:**
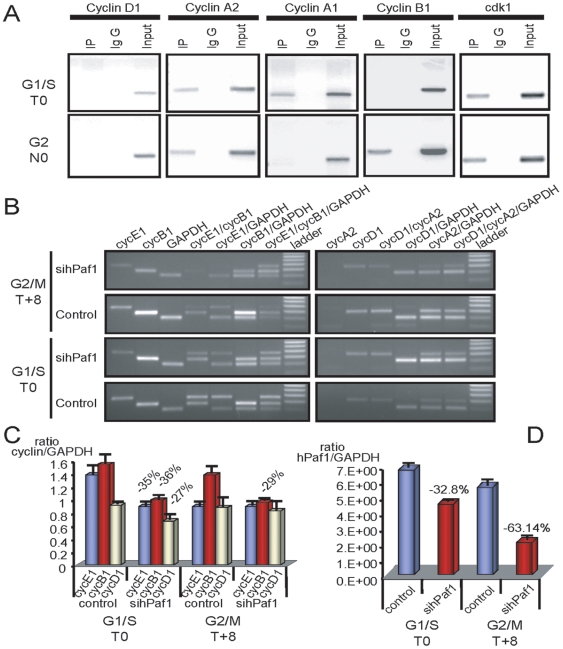
hPaf1 is a key regulator for temporal transcription of cyclins. A) Chromatine immunoprecipitation using hPaf1 anitbody for cyclins A1, A2, E1, D1, B1, and the Cdk1. An aliquot of the DNA extract before immunoprecipitation was used as a positive control for the PCR amplification. B) Analysis of cyclins E1, B1, D1, and A2 expression was done by relative competitive dropping-PCR on T0 and T+8 synchronized Panc1 cells. To measure the effect of hPaf1 expression, hPaf1 was down-regulated using the siRNA technology. C) Values of the dropping-PCR were adjusted for assay variation by dividing the integrated optical density of cyclins A2, B1, D1, and E1 mRNA by the integrated optical density of GAPDH mRNA. D) Suppression of hPaf1 expression by the Panc1 cells after transfection with siRNA oligonucleotide was measured on the same RNAs used for the dropping-PCR by one-step real time RT-PCR using a TaqMan probe following the absolute standard curve method. Standard curves were established using hPaf1 and GAPDH mimics. Sequences and conditions used in this experiment are available upon request.

**Figure 6:**
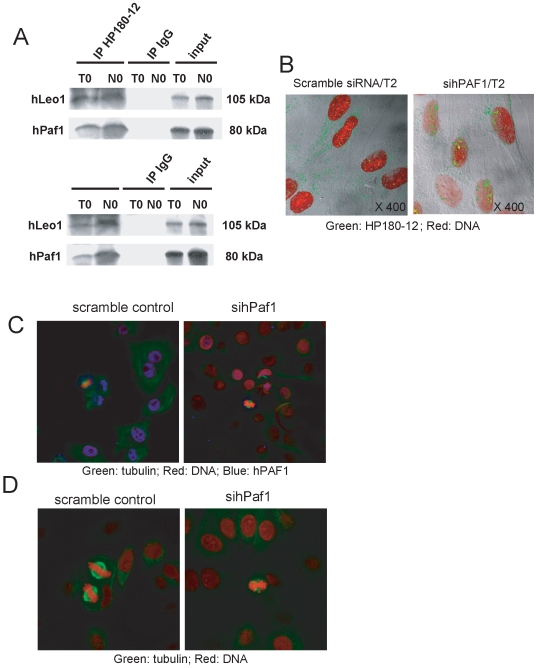
hPaf1 during initiation of DNA replication and spindle formation. A) Protein extracts from G1/S (double thymidine arrested: T0) and G2/M (thymidine/nocodazole arrested: N0) were prepared, and equal amounts of proteins were used for immunoprecipitation using HP180-12 and SJK132-20 mouse monoclonal anti-Polymerase α antibodies. Immunoprecipitates were immunoblotted with anti-hPaf1 and anti-hLeo1 antibodies. An aliquot of the protein extracts at T0 was loaded as the control expression (input). B) Fibroblast cells were arrested at the G1/S boundary and released for 12 h to reach a maximum of mitosis. The cells were analyzed for Pol α expression and sub cellular localization by confocal using HP180-12 antibody 2 h after release. At that time frame, cells invalidate for hPaf1 expression presented Pol α loaded to chromosomal DNA while cytoplasmic for the control cells. C) Invalidation of hPaf1 was evaluated by confocal microscopy. The nuclear localization of hPaf1 stained in blue using the Cy5 dye was detected for the MCF7 cells transfected by the scramble siRNA, while no staining was detected for the cells transfected with hPaf1 siRNA. D) Twenty randomly chosen cells in metaphase were observed on each slide by confocal microscopy using a FITC-labelled anti-tubulin monoclonal antibody (DM1A, Abcam). Representative pictures for MCF7 control and MCF7 invalidated for hPaf1 are presented. Eighty percent of the cells observed presented defect in tubulin polymerization and spindle formation.

The oscillatory expression pattern of hPaf1 during the cell-cycle progression lead us to hypothesize that hPaf1 is a key regulator of the cell-cycle. To identify its specific functionality during cell division, we first monitored hPaf1 influence on cell-cycle progression. For this purpose, hPaf1 was transiently invalidated by siRNA technology in synchronized Panc1 and human fibroblast cell populations. Both Panc1 and normal human fibroblast cells were arrested at the G1/S boundary by a double thymidine block. Flow cytometric analysis indicated that upto 90% and 60% of the Panc1 and fibroblast cells respectively were arrested in the G1 phase before release. The cells were then released in normal medium containing serum (time 0 = T0) and analyzed every 2 hours for 24 hours by flow cytometry for BrdU incorporation and propidium iodide staining (Fig. 4). Invalidation of hPaf1 expression in the fibroblast (Fig. 4C) and Panc1 (Fig. 5D) cells was confirmed by real-time PCR. Both Panc1 and human fibroblast cells knocked down for hPaf1 enter S-phase 2 hours in advance on an average when compared to the scramble control (measured by BrdU uptake and propidium iodide). In addition, the cells presented a longer G2-phase when hPaf1 expression was invalidated (Fig. 6B). Altogether, our results show that hPaf1 delays the onset of DNA replication by 2 hours as depicted by BrdU incorporation, but favors the G2/M transition.

### The hPaf1/hPAF complex is a key regulator of cyclin transcription initiation

At this point, one important question was whether hPaf1 was acting alone or in association with the other members of the hPAF complex. To this aim, we examined the pattern of expression of another member of the hPAF complex, hLeo1, through cell-cycle progression. For this purpose, the western blot membranes from Fig. 2C were stripped and reprobed with an anti-human Leo1 antibody. Like hPaf1, hLeo1 expression increased in S-phase and reached a maximum level of expression in G2. In mitosis, the hLeo1 level appeared comparable to its level in G1. The oscillatory pattern of expression of hLeo1 during cell-cycle progression is comparable to hPaf1 expression.

Given its role in the transcription process and its oscillatory expression pattern during cell-cycle progression, we hypothesized that hPaf1 contributes to the initiation and prolongation of cyclin expression. Binding of hPaf1 at promoter sequences of cyclin genes was investigated by chromatin immunoprecipitation using Panc1 cells that were synchronized by double thymidine or thymidine/nocodazole block. DNA and proteins were cross-linked at T0 and N0 corresponding to G1/S and early mitosis, respectively. Cross-linked hPaf1-DNA complexes, immunoprecipitated with anti-hPaf1 antibody, were analyzed (after a cross-linking reversion) for associated DNA by PCR using specific primers for the promoters of cyclins D1/A1/A2/B1, and Cdk1. The assay was also performed for the promoter of cyclin E1; however, multiple sets of primers in the promoter region were unsuccessful in the control assay for total DNA. The association of hPaf1 was specifically detected for the promoters of cyclin A1 in the G1/S phase, cyclin B1 in the G2/M phase, and cyclin A2 and Cdk1 at the G1/S and G2/M phases (Fig. 5A). No association was detected with the promoter of cyclin D1 at any stage of the cell cycle (Fig. 5A).

To monitor the effect of hPaf1 on the specific promoter activity of the aforementioned genes, a semi-quantitative dropping-RT-PCR was performed. Optimal PCR conditions required exponential amplification, which was determined by preliminary range-finding experiments. Total amplification of each multiplex reaction was kept below saturation levels to allow the amplifier to remain within the exponential range of the amplification curve and to provide semi-quantitative data. Dropping-RT-PCR was previously used and reported to monitor cyclin expression [21], [22]. The dropping-RT-PCR was carried out on RNAs extracted from cells that were synchronized by double thymidine block at T0 (G1/S) and T+8 (G2/M). Both parental and siRNA-hPaf1 knocked down Panc1 cells were investigated. Knock down of hPaf1 expression after siRNA transfection in T0 and T+8 cell populations was measured by one-step real-time PCR with a TaqMan® probe, following the absolute standard curve method. Results (ratio of hPaf1 molecules per *GAPDH* molecules) are presented in Fig. 5D. Reduction of *hPaf1* expression was 32.8% for the cell population in G1/S and 63.14% for the cells in G2/M. The same samples were used for the dropping-PCR. As compared to the control, knockdown of hPaf1 resulted in a reduction of 35%, 36% and 27% in G1/S for cyclins E1, B1, and D1, respectively (Fig. 5B, 5C). In G2/M, knockdown of hPaf1 inhibited expression only for cyclin B1 with a reduction of 29% (Fig. 5B, 5C). The level of amplification of cyclin A2 and A1 were too low to precisely measure variation in expression. Altogether, these results suggest that the hPaf1/hPAF complex contributes to regulation of cell-cycle associated cyclin transcription. Regarding cyclin D1, even though there was no observed interaction between hPaf1 and the cyclin D1 promoter, a 27% reduction in cyclin D1 expression was noted in G1/S, but not in G2/M. This suggests that hPaf1 influences cyclin D1 regulation through another mechanism. Direct effects were observed for cyclins E1 and B1, indicating that hPaf1 positively stimulates transactivation of cyclin E1 in G1/S and cyclin B1 in G1/S and G2/M. Therefore, the hPaf1/hPAF complex contributes to regulation of cell-cycle progression, and may be a key component of the cell-cycle “circadian rhythm.”

### hPaf1 plays a role during the cell-cycle independent of its role in transcription

Several lines of evidence suggest that hPaf1 possesses biological activities during the cell-cycle progression independent of its functions during transcription elongation.

First, if hPaf1 knockdown leads to 35% decrease of cyclin E expression, it also favors entry of DNA replication. These two observations seem contradictory as cyclin E is involved in the transition from G1 to S. We therefore hypothesized that hPaf1 could act in the replication process. The synthesis of the new DNA strand is the result of the collaborative work between multiple DNA polymerases: the priming DNA polymerase, α-primase (Pol α), and the replicative DNA polymerases, pol δ and pol ε. Since hPaf1 associates with the RNA polymerase II and that Pol α has a primase activity, we focused our interest on a putative interaction between hPaf1 and Pol α. Pol α is a heterotetrameric protein complex that is involved in DNA replication. The replication factor consists of the DNA synthesizing DNA polymerase α and the RNA synthesizing DNA-dependent RNA primase; both enzymes are required to synthesize the RNA/DNA primers during the initiation and elongation process of DNA replication. Proteins immunoprecipitated using both anti-polymerase α antibodies, SJK132-20 (specific for phosphorylated Pol α) and HP180-12 (specific for hypophosphorylated Pol α) [23], were analyzed for association with hPaf1 and hLeo1 by Western blot analysis. Results show that hPaf1 and hLeo1 co-immunoprecipitated with the phosphorylated and hypophosphorylated forms of Pol α (Fig. 6A). Interaction of hPaf1 and hLeo1 with Pol α was detected in G1/S and G2/M. At this point, we do not know the precise function of hPAF/Pol α interaction; however, as hPAF complex appears to delay S-phase entry, we hypothesized that hPAF1 complex/Pol α interaction enables Pol α recruitment at site of DNA replication, limiting its functionality. To confirm or invalidate this hypothesis, human fibroblast cells were treated either with hPaf1 or scramble siRNAs, synchronized at the G1/S boundary, released for 30, 60, 120, and 280 min, and analyzed by confocal analysis using the HP180-12 antibody recognizing the hypophosphorylated Pol α. Hypophosphorylated Pol α localized at replication initiation sites (Fig. 6B). Two hours after release, hypophosphorylated Pol α was mainly localized within the cytoplasm of the cells treated with scramble siRNA, while it localized at replication sites on chromosomal DNA for the cells invalidated for hPaf1 expression. Therefore, hPaf1 delays the loading of hypophosphorylated Pol α at sites of replications.

Nonetheless, the inter-chromatin accumulation of hPaf1 after nocodazole treatment reveals a direct association between spindle formation and hPaf1 subcellular localization. In addition, during prophase, hPaf1 aligns on tubule-like structures and migrates to the cellular poles at metaphase forming a crown surrounding the centrosomes. Its subcellular localization during mitosis lead us to hypothesize that hPaf1 participates in tubulin polymerization and spindle formation. To validate this hypothesis, Panc1, MCF7, and human fibroblast cells were transfected either using hPaf1 siRNA or scramble control, synchronized by double thymidine block and released for 10 and 12 hours in normal medium. Knockdown of hPaf1 was followed by confocal microscopy (Fig. 6C). As aforementioned, transient transfection of hPaf1 siRNA leads to 63% reduction of hPaf1 expression, measured by real time RT-PCR. In the current experiment, cells transfected using the scramble control revealed a strong nuclear staining of hPaf1 (Fig. 6C, blue), while no staining was detected for the cells transfected using hPaf1 siRNA. The cells were further investigated for tubulin polymerization and spindle formation by confocal microscopy. Twenty cells in metaphase were randomly chosen on each section. We observed an impairment in tubulin polymerization and spindle formation for 80% of the cells invalidated for hPaf1 expression. Representative pictures are presented on Fig. 6D for the MCF7 cells. Z-sections were performed to confirm that the defect observed was not an artifact associated with the field of observation. Defect in spindle formation may lead to the G2/M transition delay observed after hPaf1 invalidation.

## Discussion

The human homologue of the yeast transcription elongation factor, Paf1, was recently identified by our laboratory and others [24]. The role of Paf1 in transcription elongation is now well established in yeast. hPaf1 is part of the human PAF complex along with hCdc73 (parafibromin), hCtr9, and hLeo1. Interestingly, this human PAF complex lacks the Rtf1 protein, component of the yeast core complex. However, two additional subunits were recently identified as part of the human core complex, hSki8 [25] and URI [14], [26]. Both Ski8 and URI are known for their active role during RNA synthesis. Ski8 is a member of the SKI complex, involved, together with the exosome, in the 3′-5′ mRNA decay [27]. URI, unconventional prefoldin RPB5 interactor, is a scaffolding protein involved in TOR-controlled transcription pathways, controlling the transcription of distinct sets of nutrient metabolism genes [28]. These results strongly suggest a multifunctional role of the PAF complex during the transcription process, potentially involved in TOR-controlled transcription pathway [14], and events downstream of mRNA synthesis such as mRNA surveillance [11]. Additionally, both Ski8 and URI are involved in genome integrity, acting during double strand break repair in meiosis and mitosis [26], [29].

Our results present a functional multiplicity for hPaf1 acting as a key regulator of cell-cycle progression. The first line of evidence is given by the temporal expression and spatial localization of hPaf1 within the cell-cycle progression. The hPaf1 variation of expression/localization during the progression of the cell-cycle is similar to the differential expression pattern of the cyclin proteins, more specifically cyclin B1. Cyclin B1 controls the entry into mitosis as part of the maturation (or M-phase)-promoting factor (MPF). Cyclin B1 is temporally and spatially expressed with an accumulation starting in late S and a peak at the G2/M transition [30]. Its temporal accumulation is required for the G2/M transition and its degradation leads the cells to exit mitosis. Inhibition of hPaf1, using the siRNA technology, leads to a 29% decrease of cyclin B1 expression and a delay in the G2/M transition. These two lines of evidences strongly suggest that hPaf1 regulates the G2/M transition via cyclin B1 expression. However, one question remains unsolved. Why does hPaf1 align with tubulin filaments during prophase to finally form a crown surrounding the centrosome at metaphase? Invalidation of hPaf1 leads to tubulin polymerization and spindle formation defects, presenting hPaf1 as an active component during mitosis. Human Paf1 differs from its yeast homologue by its carboxyl-terminal domains, harboring a serine arginine rich and a RCC1 domains [31]. RCC1 stands for regulator of chromosome condensation and is a guanine-nucleotide-exchange factor for the small G-protein Ran [32]. Binding of RCC1 at chromatin generates high concentration of RanGTP on mitotic chromosome, favoring spindle formation [33]. Other RCC1 domain containing proteins, such as Nercc1, are also known to bind RanGTP, regulating mitotic progression [34]. In human, hPaf1 may participate in spindle formation via its RCC1 domain. Further experiments will need to be performed to confirm this hypothesis.

On the other hand, if hPaf1 knockdown favors entry of DNA replication, it also leads to 35% decrease in cyclin E expression, which seems a paradox. Cyclin E is involved in the transition from G1 to S [35]. Cyclin E is required for the assembly of the replication initiation complex, specifically for the interaction of mini-chromosome maintenance protein (MCM) replicative helicase [36] and the Pol α loading factor Cdc45 [37] onto chromosomal DNA. Increase in cyclin E expression favors loading of Cdc45 at DNA replication initiation site and promotes DNA replication. Recently, another laboratory observed similar results on DNA replication after hPaf1 invalidation. Yart et al. [14] reported that siRNA-mediated knockdown of parafibromin and hPaf1 on ansynchronously growing HeLa and U2-OS cell populations induced entry into S phase, validating our data. This apparent contradiction among the results points out a multiplicity of function for hPaf1 during the G1/S transition. On one hand, hPaf1 acts through cyclin E to initiate DNA replication, but on the other hand, it acts through an unknown mechanism to delay DNA replication. As aforementioned, the two newly identified hPaf1 partners, Ski8 and URI, function during DNA replication for DNA double strand break repair. This leads us to hypothesize that hPaf1 is involved in DNA repair. In addition, our results show that hPaf1 interacts with both hypo and phosphorylated forms of the DNA polymerase-α primase leading to an apparent sequestration of Pol α within the cytoplasm of the cells. Doing so, hPaf1 might act as a sensor allowing all proteins required for proper replication to be actively present at the same time.

**Figure 7:**
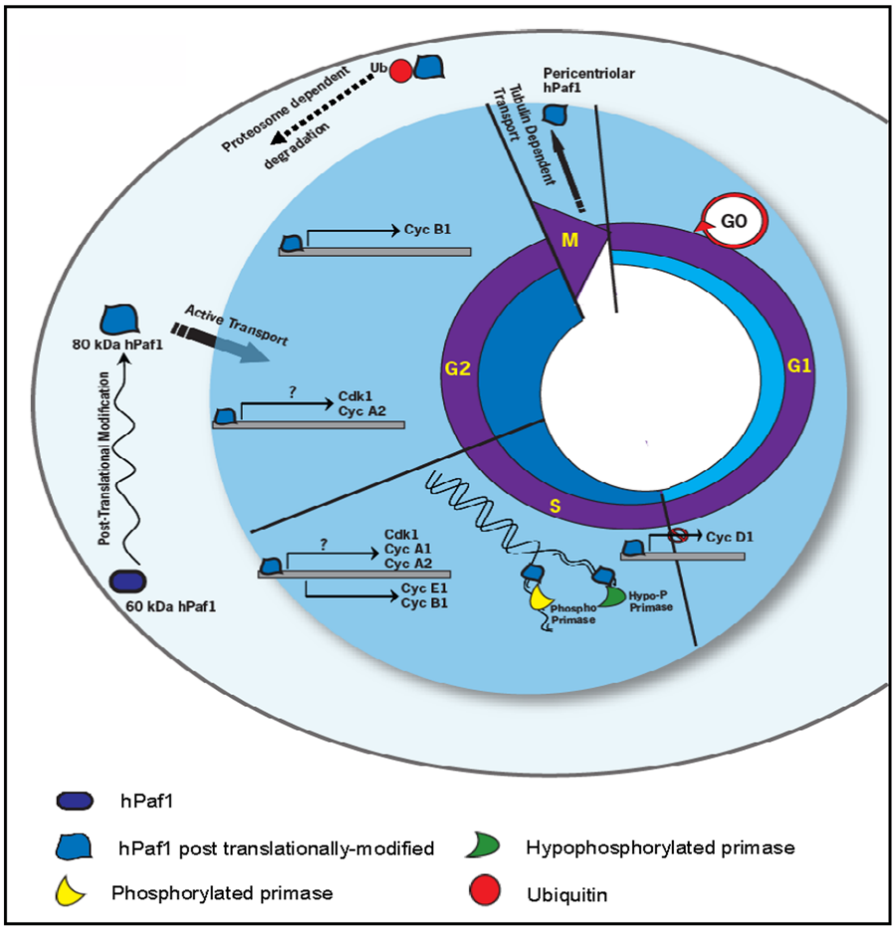
hPaf1 during cell cycle progression. hPaf1 expression initiates in early G1, increases during the S-phase and reaches a maximum level in early G2. Synthesized as a 60 kDa protein, hPaf1 translocates as an 80 kDa protein into the nucleus. In the nucleus, hPaf1 directly regulates the expression of the cyclins A1, A2, D1, E1, B1. In S phase, hPaf1 interacts with the DNA polymerase α-primase complex and delays the onset of DNA replication. At mitosis, hPaf1 migrates to the cellular pole, forming a crown surrounding the centrosomes. It is then possibly shuttled via tubulin filaments to be degraded by the proteasome.

In light of the results presented in this article and summarized in Fig. 7, hPaf1 and/or hPAF complex controls the cell-cycle progression. Our results suggest a dual functionality for the members of the hPAF complex. Partial invalidation of hPaf1 promotes the onset of DNA replication, presenting hPaf1 as a tumor suppressor, while overexpression of hPaf1 presents a transforming phenotype. A tight temporal and spatial regulation of the hPAF complex members may be required for proper functionality, with loss of stoichiometry or mutation of at least one of the members potentially leading to cancer development.

## Materials and Methods

### Materials

The Panc1 and CD18/HPAF cell lines [20], was obtained from the American Type Culture Collection (ATCC). The human fibroblast cells were a gift from the Dr. Michel Ouellette (Eppley cancer Institute, Omaha, NE). For double thymidine cell arrest, cells were treated with thymidine (Sigma) at 2 mM final concentration for 18 hours, released in normal medium, and treated again for 18 hours with a 2 mM final concentration of thymidine. For the thymidine/nocodazole (Sigma) block, cells were incubated with thymidine at a 2 mM final concentration for 18 hours, released in normal medium for 10 hours and incubated with nocodazole at 100 nM final concentration for 15 hours. The proteasome inhibitor MG132 was purchased from BIOMOL international. The mouse monoclonal antibody anti-hPaf1 was produced in our laboratory. The rabbit polyclonal antibody anti-hLeo1 was provided by Dr. Danny Reinberg (Howard Hughes Medical Institute, New York, NY). Both mouse monoclonal anti-polymerase α antibodies SJK132-20 and HP180-12, were provided by Dr. Irena Dornreiter (Heinrich-Pette-Institute, Hamburg, Germany). The rabbit polyclonal anti-ubiquitin antibody was purchased from Calbiochem and the JLA-20 rabbit polyclonal anti-actin antibody from the University of Iowa.

### Flow cytometry

Confluent cells were detached from the flask with trypsin, washed in PBS, and fixed in 2 mM glycin [pH 2]/70% ethanol. Fixed-cells were then washed and resuspended in 1 ml of Telford reagent (90 mM EDTA, 2.5 mU of RNase A/ml, 50 µg of propidium iodide/ml, and 0.1% Triton X-100 in PBS). The total DNA content was analyzed in a FACSCalibur analyzer using ModFit LT software (Becton Dickinson).

### Confocal Microscopy

Cells were plated onto sterile round cover slips (CIR 18-1 Fisher brand 12-545-10) and grown in 12-well plates. Cells were fixed in acetone/methanol (1∶1; pre-chilled to −20°C) and permeabilized in 0.1% Triton X-100 in PBS. Cells were washed in PBS and incubated with primary (for 2 hours) and secondary antibodies (for 30 min) at room temperature. Antibodies were diluted in 5% goat serum.

### Preparation of cytoplasmic and nuclear protein extract, immunoprecipitation, and western blot

The protocol for cytoplasmic and nuclear extract preparation was adapted from previously published procedures [38], [39]. Cells were allowed to swell on ice for 30 minutes in hypotonic cytoplasmic extraction buffer (10 mM Hepes, pH 7.4, 10 mM KCl, 0.2% NP-40, 0.1 mM EDTA, 10% glycerol, 1.5 mM MgCl_2_, 1 mM DTT, 1 mM PMSF, 5 mM Na_3_VO_4_, 5 mM NaF), supplemented with complete protease inhibitor cocktail [Roche]). Cell disruption was accomplished by several passages through a 25G needle. Nuclei were collected by centrifugation at 16,000 g, and the supernatant further centrifuged at 16,000 g to yield the final cytoplasmic extract. Nuclear pellets were incubated in hypertonic nuclear extraction buffer (20 mM Hepes, [pH 7.6], 420 mM NaCl, 1 mM EDTA, 20% glycerol, 1.5 mM MgCl_2_, 1 mM DTT, 1 mM PMSF, 5 mM Na_3_VO_4_, 5 mM NaF, supplemented with complete protease inhibitor cocktail [Roche]). Insoluble materials were precipitated by centrifugation. The supernatant was collected and used as nuclear extract.

Immunoprecipitation was performed by adding 5 µg of the appropriate antibodies, and negative controls IgG control to 100 µg of protein extract. The reaction mixture was incubated overnight on a rotator at 4°C, pulled down with protein G beads, and washed in TBST. Immuno-complexes were eluted from the protein G bead by boiling in 6x loading buffer. Immunoprecipitate proteins or protein extracts were resolved on a 10% SDS gel and transferred onto polyvinylidene difluoride (PVDF) membranes. Blocked membranes were reacted with anti-hPaf1 mouse monoclonal antibody at 1 µg/ml in PBS containing 0.05% Tween-20 (PBST) overnight at 4°C. Membranes were then washed six times (ten minutes each) in PBST and incubated in TBST containing sheep anti-mouse polyclonal antibody conjugated to horseradish peroxidase (1∶2000 dilution) for one hour at room temperature. Following six rinses in PBST, ECL reagents (Amersham, USA) were applied to membranes.

### ChIP Assay

Panc1 cells were fixed with 1% formaldehyde, washed, harvested and re-suspended in 200 µl SDS lysis buffer (1% SDS, 10 mM EDTA, 50 mM Tris-HCl [pH 8.1], 1 mM PMSF, and 1 µg/ml aprotinin). Samples were sonicated and diluted in ChIP Dilution Buffer (0.01% SDS, 1.1% Triton X-100, 1.2 mM EDTA, 16.7 mM Tris-HCl [pH 8.1], 167 mM NaCl, 1 mM PMSF, and 1 µg/ml aprotinin). Immunoprecipitation was performed as previously described. Chromatin extracts were pulled down with a protein G bead. The samples were washed extensively and the cross-links were reversed by incubating the samples at 65°C for 4 hours in 0.3 M NaCl. Immunoprecipitated DNA was amplified by PCR with specific primers for cyclins A1, A2, E1, D1, B1, and the Cdk1, loaded on a 2% agarose gel and visualized with ethidium bromide. All primers used in this study are available upon request.

### Dropping-RT-PCR

Total cellular RNA from Panc1 cells was extracted using the RNAeasy kit (Qiagen) and processed for reverse transcription and primer-dropping PCR. Primer sequences and amplification conditions have been described previously[22]. The initial PCR activation step was at 94°C for four minutes, followed by the denaturation step at 94°C for one minute, primer-annealing step at 55°C for 30 seconds, extension step at 72°C for one minute, and the final extension step at 72°C for ten minutes. PCR reaction products were subjected to electrophoresis using a 2% agarose gel. Gels were stained using 0.5 µg/ml of ethidium bromide, illuminated with UV light, photographed using a digital camera, and analyzed by computerized densitometric scanning of the images using QuantityOne version 4.1.1 software (Bio-Rad). Values were adjusted for assay variation by dividing the integrated optical density of cyclins A2, B1, D1, and E1 mRNA by the integrated optical density of GAPDH mRNA.

### RNA interference

The region of hPaf1 targeted for siRNA was 5′-AACAGGUUCGUCCAGUACAAA-3′. Synthetic sense and antisense oligonucleotides (Dharmacon, Lafayette, CO) were annealed in 100 mM potassium acetate, 30 mM HEPES-KOH (pH 7.4), and 2 mM magnesium acetate for one minute at 90°C and one hour at 37°C, and frozen. Oligonucleotides were transfected into cells with *Trans*IT-TKO (Mirus, Madison, WI) in accordance with the supplier's recommendations. Cells were treated by double thymidine incorporation 24 hours after transfection when required.
